# Altered cytoskeletal arrangement in induced pluripotent stem cells and motor neurons from patients with riboflavin transporter deficiency

**DOI:** 10.1242/dmm.046391

**Published:** 2021-02-24

**Authors:** Alessia Niceforo, Chiara Marioli, Fiorella Colasuonno, Stefania Petrini, Keith Massey, Marco Tartaglia, Enrico Bertini, Sandra Moreno, Claudia Compagnucci

**Affiliations:** 1 Department of Science, Laboratorio Interdipartimentale di Microscopia Elettronica, University Roma Tre, Rome 00146, Italy; 2 Department of Neuroscience, Unit of Neuromuscular and Neurodegenerative Diseases, Laboratory of Molecular Medicine, Istituto di Ricovero e Cura a Carattere Scientifico (IRCCS) Ospedale Pediatrico Bambino Gesù, Rome 00146, Italy; 3 Genetics and Rare Diseases Research Division, IRCCS Ospedale Pediatrico Bambino Gesù, Rome 00146, Italy; 4 Confocal Microscopy Core Facility, Research Laboratories, IRCCS Ospedale Pediatrico Bambino Gesù, Rome 00146, Italy; 5 Science Director, Cure RTD Foundation, 6228 Northaven Road, Dallas, TX 75230, USA

**Keywords:** Riboflavin transporter deficiency, IPSCs, Motor neurons, Tubulin, Riboflavin, NAC

## Abstract

The cytoskeletal network plays a crucial role in the differentiation, morphogenesis, function and homeostasis of the nervous tissue, so that alterations in any of its components may lead to neurodegenerative diseases. Riboflavin transporter deficiency (RTD), a childhood-onset disorder characterized by degeneration of motor neurons (MNs), is caused by biallelic mutations in genes encoding the human riboflavin (RF) transporters. In a patient-specific induced pluripotent stem cells (iPSCs) model of RTD, we recently demonstrated altered cell-cell contacts, energy dysmetabolism and redox imbalance. The present study focuses on cytoskeletal composition and dynamics associated to RTD, utilizing patients' iPSCs and derived MNs. Abnormal expression and distribution of α- and β-tubulin (α- and β-TUB), as well as imbalanced tyrosination of α-TUB, accompanied by an impaired ability to re-polymerize after nocodazole treatment, were found in RTD patient-derived iPSCs. Following differentiation, MNs showed consistent changes in TUB content, which was associated with abnormal morphofunctional features, such as neurite length and Ca^2+^ homeostasis, suggesting impaired differentiation. Beneficial effects of RF supplementation, alone or in combination with the antioxidant molecule N-acetyl cystine (NAC), were assessed. RF administration resulted in partially improved cytoskeletal features in patients' iPSCs and MNs, suggesting that redundancy of transporters may rescue cell functionality in the presence of adequate concentrations of the vitamin. Moreover, supplementation with NAC was demonstrated to be effective in restoring all the considered parameters, when used in combination with RF, thus supporting the therapeutic use of both compounds.

## Abstract (precis)

**Summary:** This study deals with pathomechanisms underlying riboflavin transporter deficiency, a rare recessively inherited early-onset neurodegenerative condition. Using patient-derived iPSCs, we report on cytoskeletal abnormalities, which are reverted by combined riboflavin/antioxidant treatment.

## INTRODUCTION

Riboflavin (RF), or vitamin B2, as a precursor of flavin mononucleotide (FMN) and flavin adenine dinucleotide (FAD), is a crucial micronutrient. In humans, this compound is acquired by the diet (McCormick, 1989), and absorbed through three specific transporters, SLC52A1, SLC52A2 and SLC52A3 (herein referred to as RFVT1, RFVT2 and RFVT3), differently distributed in human tissues (Gallai et al., 1981; Bosch et al., 2012; Udhayabanu et al., 2017).

Riboflavin transporter deficiency (RTD), also referred to as Brown–Vialetto–Van Laere syndrome (BVVL), is a rare childhood-onset disorder, characterized by progressive muscle weakness, bulbar dysfunction, sensorineural hearing loss, optic atrophy, sensory ataxia and respiratory compromise (Van Laere, 1966; O'Callaghan et al., 2019). This progressive motor neuron (MN) disorder, often leading to death in the first years of life, is inherited as an autosomal recessive trait. Biallelic *SLC52A3* or *SLC52A2* mutations have been identified as causative of the disorder (Green et al., 2010; Johnson et al., 2012). It is unclear whether the disease may also be inherited as an autosomal dominant trait with incomplete penetrance (Van Laere, 1966; Brucher et al., 1981; Hawkins et al., 1990; De Grandis et al., 2005), although this has not been confirmed after the genetic characterization (Johnson et al., 2012).

As inadequate RF levels are associated with this syndrome, high-dose vitamin supplementation is likely to result in amelioration of the clinical phenotype. Indeed, patients treated with a dose of 10 mg/kg/day show improved muscle strength, motor function, respiration, hearing and vision (Bosch et al., 2012).

Unfortunately, *in vivo* models for RTD are of little help in understanding the molecular pathogenesis of the disease. In fact, the two independently generated mouse models show embryonic or perinatal lethality, preventing their use for mechanistic studies (Intoh et al., 2016; Yoshimatsu et al., 2016).

The development of the nervous system requires cytoskeleton-mediated processes, coordinating neuronal migration and differentiation. Specifically, microtubules (MTs) are required for proper neural stem cell/precursor proliferation, polarity and migration. The arrays of axons and dendrites of MTs provide a structural backbone that allows them to acquire and maintain their specialized morphologies, while ensuring efficient intracellular trafficking for proteins and organelles. Proper functioning of MTs and their interacting and regulatory proteins is crucial for the health of the nervous system. In particular, MTs are crucial for stabilizing the axon structure and modulating axon elongation (Yamada et al., 1970; Baas et al., 2016). Accordingly, abnormalities of the MT systems of axons and dendrites have been recognized as a major contributor to neurodegenerative diseases (Matamoros and Baas, 2016). MTs, which are composed of α- and β-tubulin (α- and β-TUB), are dynamic structures characterized by continuous polymerization/depolymerization cycles (Galjart, 2010), mostly regulated by a different distribution of the stable (de-tyrosinated, Detyr-TUB) and dynamic (tyrosinated, Tyr-TUB) forms of tubulin (Gundersen et al., 1987; Gadadhar et al., 2017). Cytoskeletal proteins are highly sensitive to oxidation, because their supra-molecular organization depends on the presence of exposed sulphydryl residues (Piermarini et al., 2016). This particularly applies to MTs, the dynamics of which are influenced by reactive oxygen species (ROS) (Islam et al., 2016), thus making them good candidate targets for the oxidative-mediated neuronal injury underlying axonal degeneration (Wilson and González-Billault, 2015). Axons are indeed susceptible to oxidative damage, as their glutathione (GSH) content is lower than total GSH content of the parent cell body (Romero et al., 1991; Wilson and González-Billault, 2015). Accordingly, administration of oxidized glutathione (GSSG) interferes with the dynamic form of tubulin in cultured MNs (Carletti et al., 2011). The correlation between axonal integrity and GSSG/GSH levels may explain why in several neuropathies, distal to proximal axon degeneration occurs long before, or even in the absence of, cell death (Piermarini et al., 2016). In this context, it should be mentioned that RF exerts an antioxidant function, through its conversion of reduced to oxidized form, and as a component of the GSH redox cycle (Ashoori and Saedisomeolia, 2014).

The induced pluripotent stem cells (iPSCs) technology provides a novel experimental tool to investigate the pathophysiology of rare diseases affecting the nervous system, which is not readily accessible in humans (Compagnucci et al., 2014). Consistent with our recent work (Colasuonno et al., 2020), we took advantage of this approach, using iPSCs obtained from fibroblasts of RTD patients, to further characterize molecular and cellular aspects of the disease. Based on our previous results, demonstrating abnormal cell morphology, cell-cell contacts and organelle dysfunction, we focused on the cytoskeleton, the dynamics of which is fundamental to ensure proper intercellular communication and intracellular trafficking (Compagnucci et al., 2014), and explored pathophysiological aspects of RTD using iPSCs and iPSC-derived MNs as *in vitro* model systems. Possible beneficial effects of RF/antioxidant treatments were also investigated to suggest novel therapeutic approaches to RTD. Among the possible ROS scavengers, we selected the GSH precursor N-acetyl cystine (NAC), considering that RF deficiency likely compromises the maintenance of proper GSSG/GSH levels.

## RESULTS

### Characterization of RTD patient-derived iPSCs

**Fig. 1.**
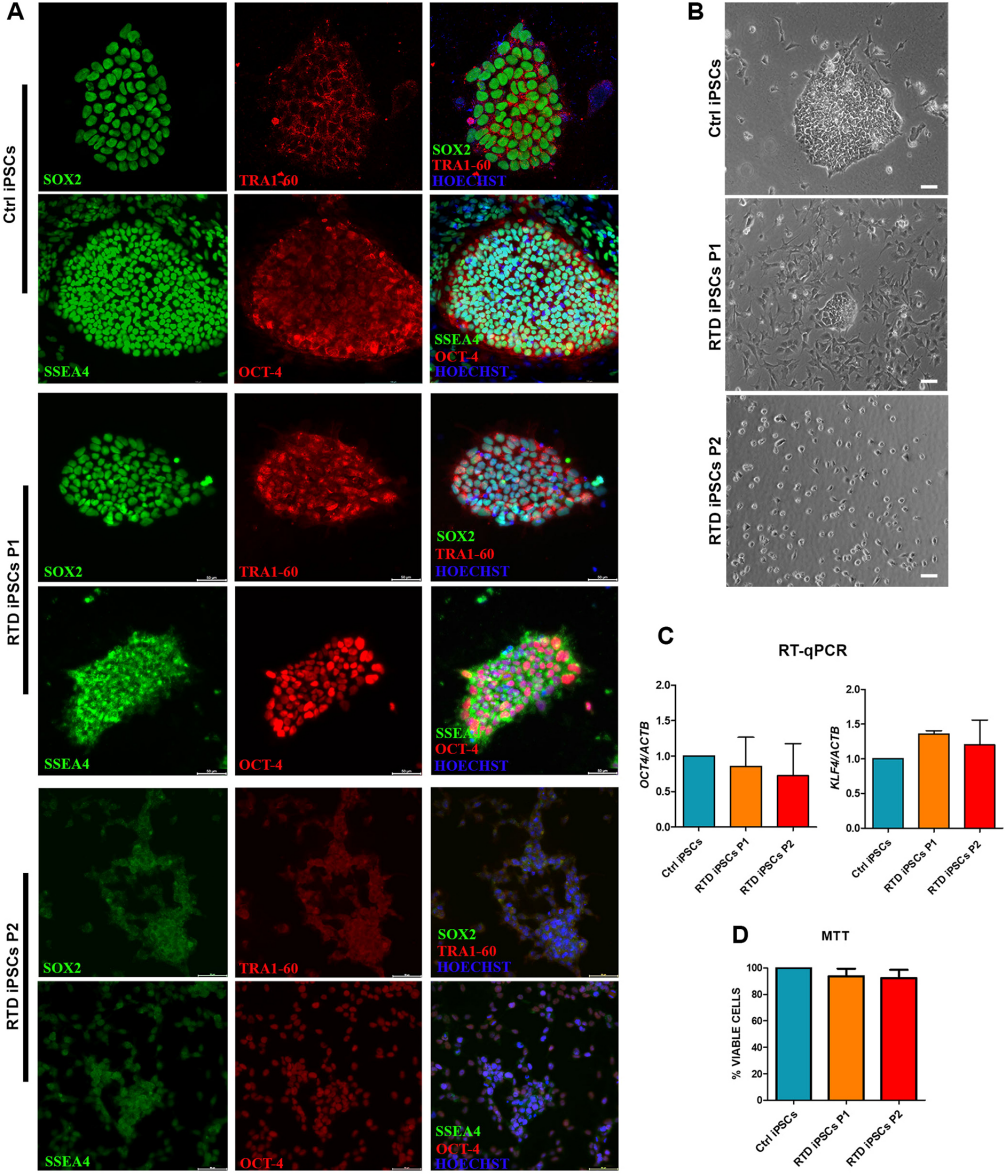
**Patient-specific model validation and characterization of RTD iPSCs.** (A) iPSCs from two RTD patients express pluripotentcy markers, as assessed by immunofluorescence for SSEA4, SOX-2 (in green), OCT-4 and TRA 1-60 (in red). (B) Phase contrast images show an absence of typical colonies in RTD iPSCs compared to Ctrl. (C) *OCT4* and *KLF4* gene expression evaluated by RT-qPCR, using ACTB as a reference standard. Similar values of Ctrl, RTD P1 and RTD P2 iPSCs are detected. (D) MTT assay for cell viability. The percentage of viable cells in P1 and P2 iPSC cultures are comparable to Ctrl. Data are mean±s.e.m. Scale bars: 50 μm (A); 20 μm (B).

iPSCs were obtained from skin biopsies of two patients (P1 and P2) with mutations in *SLC52A2*. Control (Ctrl) iPSCs were obtained from two healthy individuals. As the results obtained from Ctrl cells were consistently similar, we show representative images from either samples. To confirm the pluripotency of our cell model (see Materials and Methods), we confirmed their positivity to the pluripotency markers SOX2, TRA-1-60, SSEA4 and OCT4 (also known as POU5F1) by immunofluorescence, together with RT-qPCR analyses of *OCT4* and *KLF4* gene expression (Fig. 1A,C). The RT-qPCR documented statistically non-significant differences of either pluripotency marker among Ctrl and RTD iPSCs (Fig. 1C). Based on the essential role of RF in flavoprotein functionality, we investigated the activity of succinate dehydrogenase, a mitochondrial enzyme, the function of which directly depends on vitamin B2. Incubating iPSCs with the tetrazolium salt MTT, we evaluated the ability of succinate dehydrogenase to metabolize this salt in iPSCs. In particular, MTT reduction leads to the formation of formazan. As viable cells, unlike dying ones, reduce MTT, the amount of the formazan produced was proportional to the number of viable cells (Fig. 1D). The analysis provided evidence that no significant change in cell survival is associated with RTD, and that no gross perturbation of mitochondrial flavoenzymatic function occurs in RTD, at least in the tested *in vitro* conditions. Morphological analyses, while confirming the ability of Ctrl iPSCs to form large roundish colonies with regular edges, documented that RTD patient-derived iPSCs were characterized by smaller irregularly shaped colonies (P1), or even individual cells, separated from each other (P2) (Fig. 1B).

### Altered expression and distribution of α- and β-TUB in RTD iPSCs

**Fig. 2.**
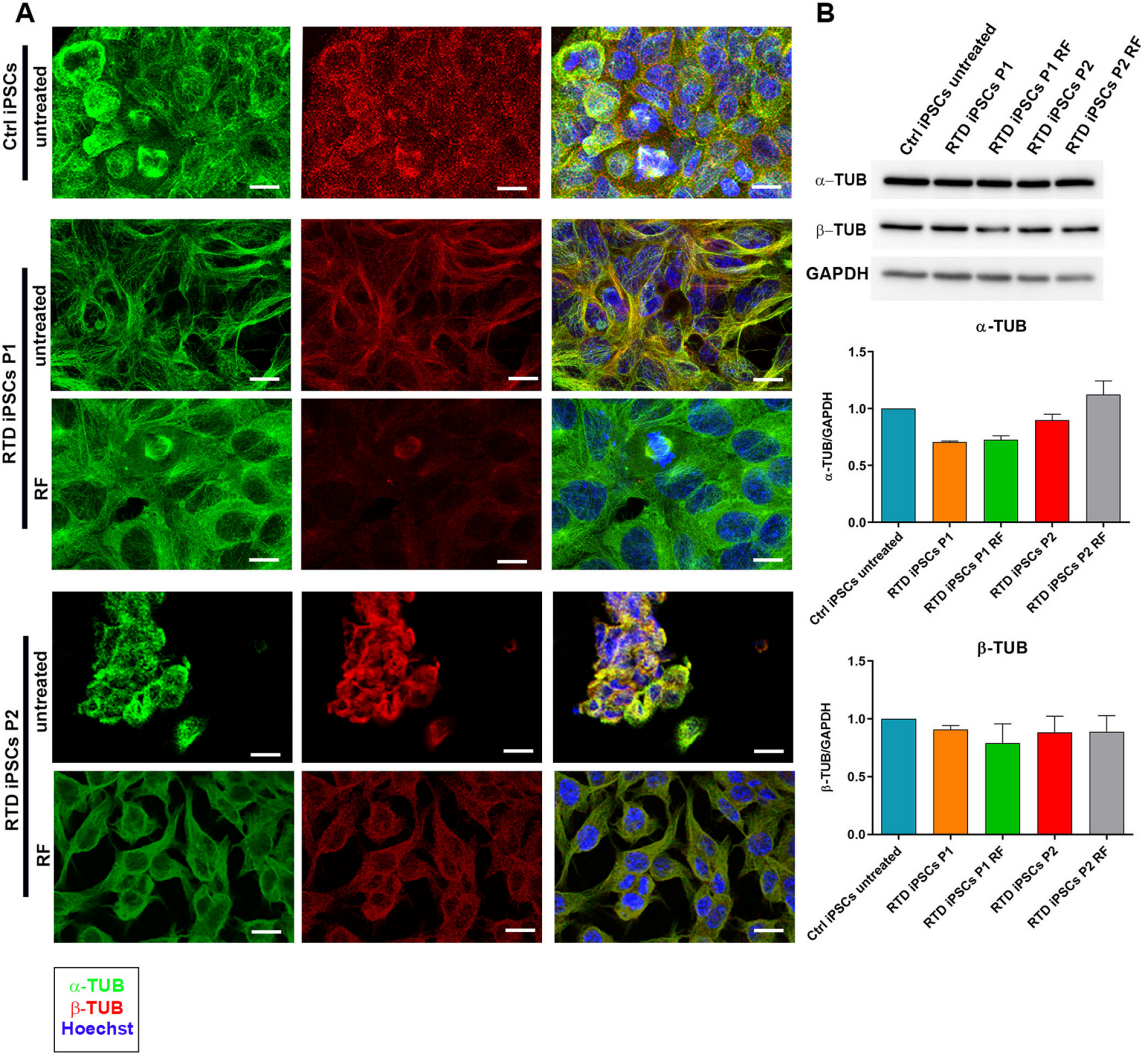
**Altered intracellular distribution of α- and β-TUB in RTD iPSCs.** (A) Immunofluorescence images of α-TUB (in green) and β-TUB (in red), demonstrating different intracellular distribution in RTD iPSCs compared to Ctrl cells. Both MT components aggregate in the perinuclear area of RTD P2 cells. Nuclei are stained with Hoechst (in blue). After RF treatment, the distribution patterns of both markers improve, becoming similar to those of Ctrl. Scale bars: 10 μm. (B) Western blot analyses showing the expression levels of α-TUB, β-TUB and GAPDH as loading control performed on protein extracts of Ctrl and RTD iPSCs with and without RF supplementation. The bar graphs below show the quantification of α-TUB and β-TUB relative to GAPHD levels, and data are mean±s.e.m. of three experiments. According to a Kruskal–Wallis test, the medians of the α-TUB/GAPDH quantification vary significantly (*P*=0.0002).

As patients' iPSCs fail to form well-defined round-shaped colonies, we decided to further characterize their cytoskeletal architecture. We focused on MTs, as alterations to these structures or to MT-associated or MT-regulating proteins may impair neuronal morphology and function (Gonçalves et al., 2018; Binarová and Tuszynski, 2019). To this aim, we investigated the distribution and levels of α-TUB and β-TUB, which are major components of microtubules (Fig. 2). Immunofluorescence analyses of both cytoskeletal markers revealed RTD-associated abnormal distribution, particularly in P2 iPSCs, in which a patchy and mostly perinuclear localization for both α-TUB and β-TUB (Fig. 2A) was observed. In addition, α-TUB and β-TUB signals were slightly lower in RTD cells compared to Ctrl iPSCs, as confirmed by western blotting and its corresponding quantification (Fig. 2B). These data have also been confirmed by the quantification of fluorescence intensity signal for α-TUB and β-TUB fluorescence relative to cell number per frame performed on 20× microscopic images (Fig. S1).

To assess whether RF supplementation is efficacious in restoring a ‘healthy’ phenotype in RTD cells, we performed RF treatments and observed rescue of the RTD condition, by recovering the localization pattern and the expression levels of α-TUB in P2 (Fig. 2A,B).

### Post-translational modifications of α-TUB in RTD iPSCs

**Fig. 3.**
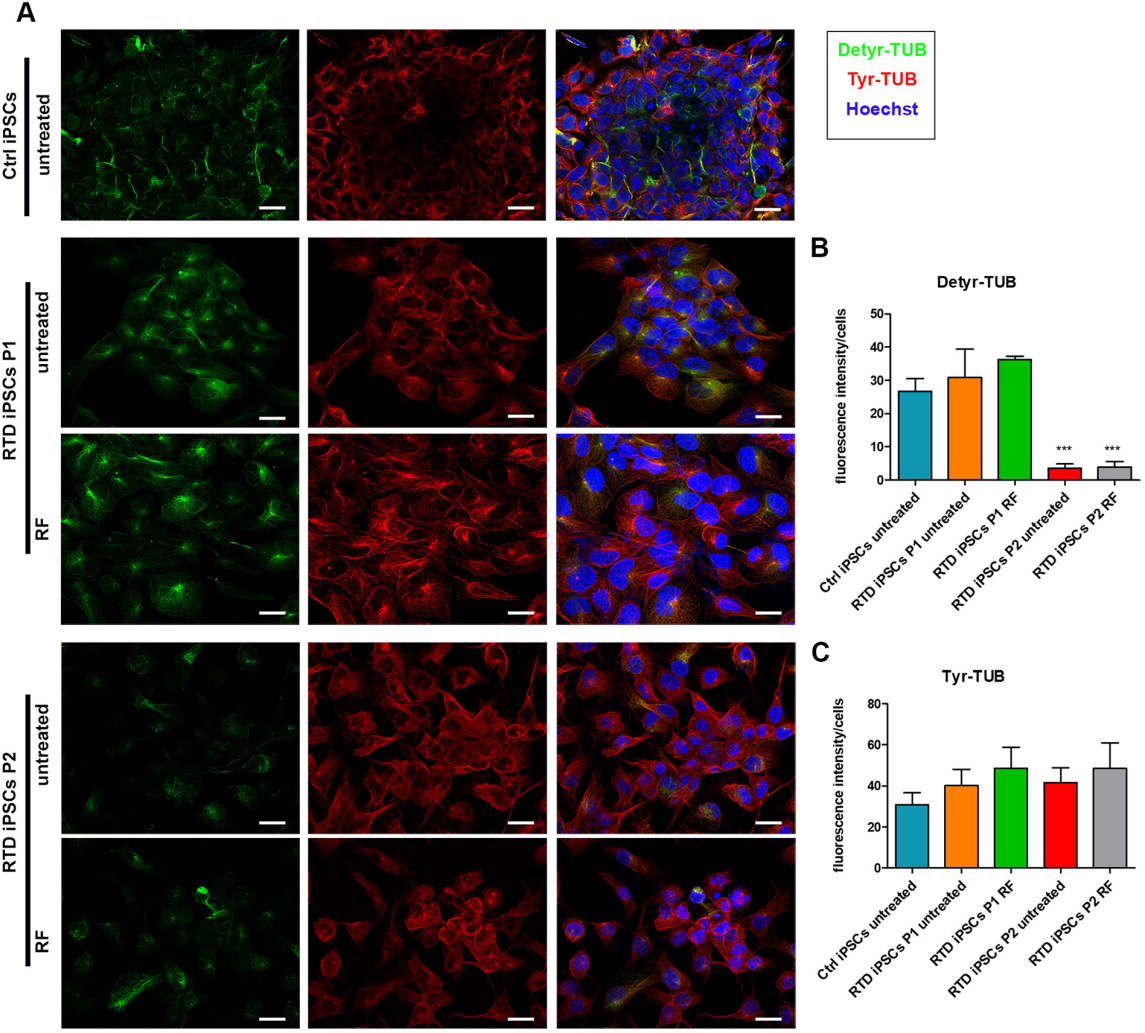
**Altered MT dynamics in RTD iPSCs** **are** **revealed by α-TUB tyrosination/detyrosination cycle analysis.** (A) Immunofluorescence images of Detyr-TUB (in green) and Tyr-TUB (in red), demonstrating abnormal concentration of the two markers in the perinuclear region of RTD iPSCs. Nuclei are stained with Hoechst (in blue). Scale bars: 10 μm. (B) Bar graph of Detyr-TUB fluorescence intensity and statistical analysis, revealing dramatically decreased levels of Detyr-TUB in RTD P2 iPSCs (***P≤0.001 according to non-parametric ANOVA tests). (C) Bar graph of Tyr-TUB fluorescence intensity and statistical analysis of fluorescence intensity, revealing non-significant differences in Tyr-TUB among Ctrl and RTD iPSCs.

To get a further insight into MT dynamics, we decided to investigate the localization and intensity of the tyrosinated and detyrosinated-TUB (Tyr- and Detyr-TUB, respectively) by immunofluorescence analysis. It is known that post-translational modifications of tubulins are used to unveil stable versus labile microtubules, therefore we studied the tyrosination/detyrosination cycle, occurring at the C-terminal end of α-TUB (Peris et al., 2006). To investigate post-translational modifications of α-TUB, we performed quantitative analysis of the fluorescence intensity, using anti-Tyr-TUB and anti Detyr-TUB. Although both signals were uniformly distributed throughout the cytoplasm of Ctrl cells, these markers were concentrated in the perinuclear region in both patients' iPSCs (Fig. 3A). We also observed a dramatic decrease in Detyr-TUB in RTD P2 iPSCs, even though this was associated with increased Tyr-TUB levels (Fig. 3B,C). Levels and distribution of Detyr- or Tyr-TUB remained altered in patients' iPSCs, even after RF supplementation, proving that such treatment is unable to rescue the altered phenotype involving post-translational modifications of α-TUB (Fig. 3A-C).

### Analysis of cytoskeletal dynamics in iPSCs after nocodazole treatment

**Fig. 4.**
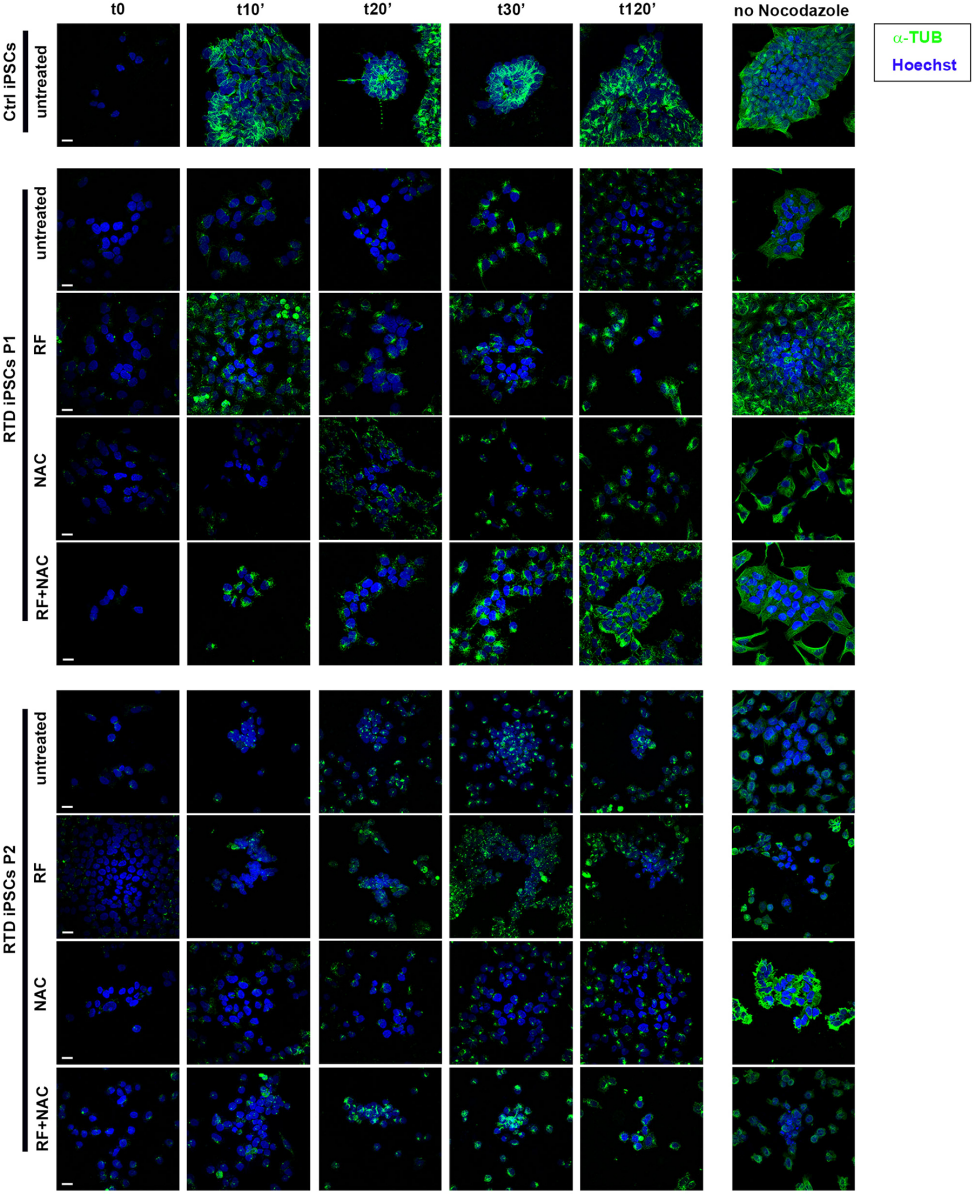
**Defective RFVT2 function affects MT dynamics, but pre-treatment with RF plus NAC is able to rescue the altered phenotype.** Immunofluorescence analyses showing reduced re-polymerization of MTs (labeled for α-TUB, green) after nocodazole treatment in RTD iPSCs, as compared to Ctrl cells. Ctrl and RTD iPSCs were also treated with RF, NAC and RF plus NAC, before nocodazole treatment at t0 min, t10 min, t20 min, t30 min and t120 min. The right column shows corresponding cell cultures not undergoing nocodazole treatment. Immunofluorescence staining with α-TUB (in green) demonstrates a markedly delayed MT re-polymerization in patient iPSCs. Nuclei are stained with Hoechst (in blue). Scale bars: 10 μm.

**Fig. 5.**
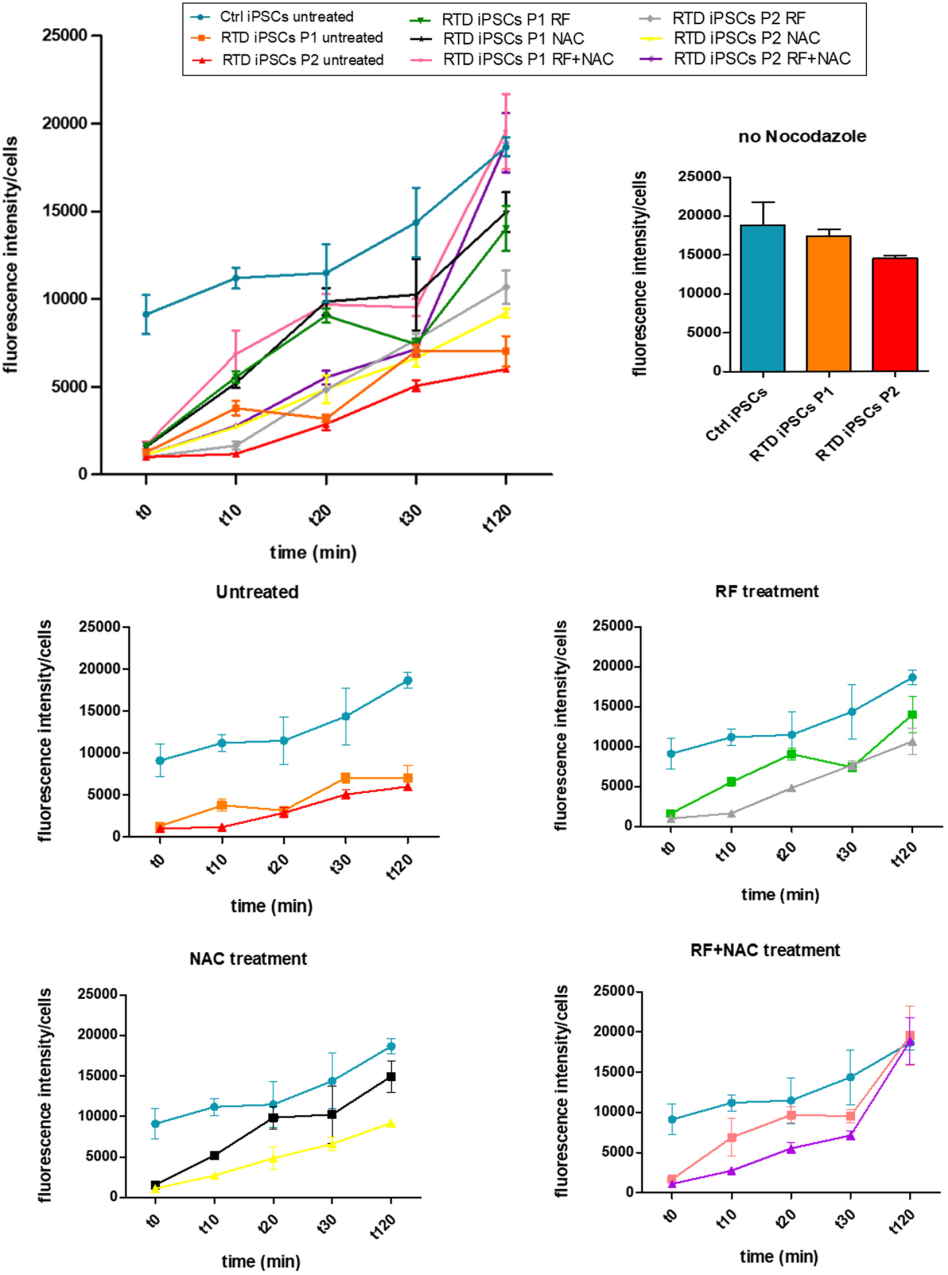
**Graphical representation of defective MT re-polymerization in RTD iPSCs rescued by pre-treatment with RF plus NAC.** Timecourse fluorescence analysis (t0 min, t10 min, t20 min, t30 min, t120 min) showing Ctrl and RTD iPSCs treated with RF, NAC and RF plus NAC, before nocodazole treatment. Both RF and NAC alone produce a significant improvement in the speed of MTs re-polymerization in RTD iPSCs. RF plus NAC combined treatment led to an even more rapid and efficient re-polymerization, with a similar pattern to Ctrl iPSCs. Data are mean±s.e.m.

To shed further light on the MT dynamics, we treated Ctrl and patients' iPSCs with the tubulin-depolymerising agent nocodazole to monitor the re-polymerization ability and timing of MTs on RTD and Ctrl iPSCs. Following drug administration, MT regrowth was analysed in timecourse experiments (t0 min, t10 min, t20 min, t30 min and t120 min). Immunofluorescence staining with α-TUB (Fig. 4) demonstrated efficient re-polymerization in Ctrl iPSCs as early as 10 min after nocodazole washout, with complete recovery of properly polymerized MTs at 120 min. Quantification analysis revealed a linear increase of α-TUB re-polymerization (Fig. 5). By contrast, MT re-polymerization appeared to be markedly delayed in both patients' iPSCs, in which α-TUB levels were lower than in Ctrl iPSCs, at any time point considered (Figs 4,5). When RTD cells were pretreated with RF, a significant improvement in the speed of MT re-polymerization was observed. Specifically, in P1 cells this event started earlier (t10 min) than in untreated cells and proceeds consistently, whereas in P2 cells the process was still delayed, reaching higher levels of α-TUB polymerization at later time points (t30 min) (Fig. 5). As RF treatment was unable to rescue the phenotype in RTD iPSCs in P2, we were prompted to investigate whether an antioxidant molecule could further rescue the RTD phenotype, particularly in combination with RF. We tested the effect of the GSH precursor NAC, which demonstrably accelerates re-polymerization of α-TUB in patients' iPSCs. This occurs from 20 min (P1) or 30 min (P2) after nocodazole washout and increasing thereafter (Figs 4,5). Finally, when combining RF and NAC treatments, patients' iPSCs show more rapid and more efficient α-TUB re-polymerization, similarly to Ctrl iPSCs (Fig. 5).

### Morphological abnormalities of RTD MNs and rescue by RF and NAC treatments

**Fig. 6.**
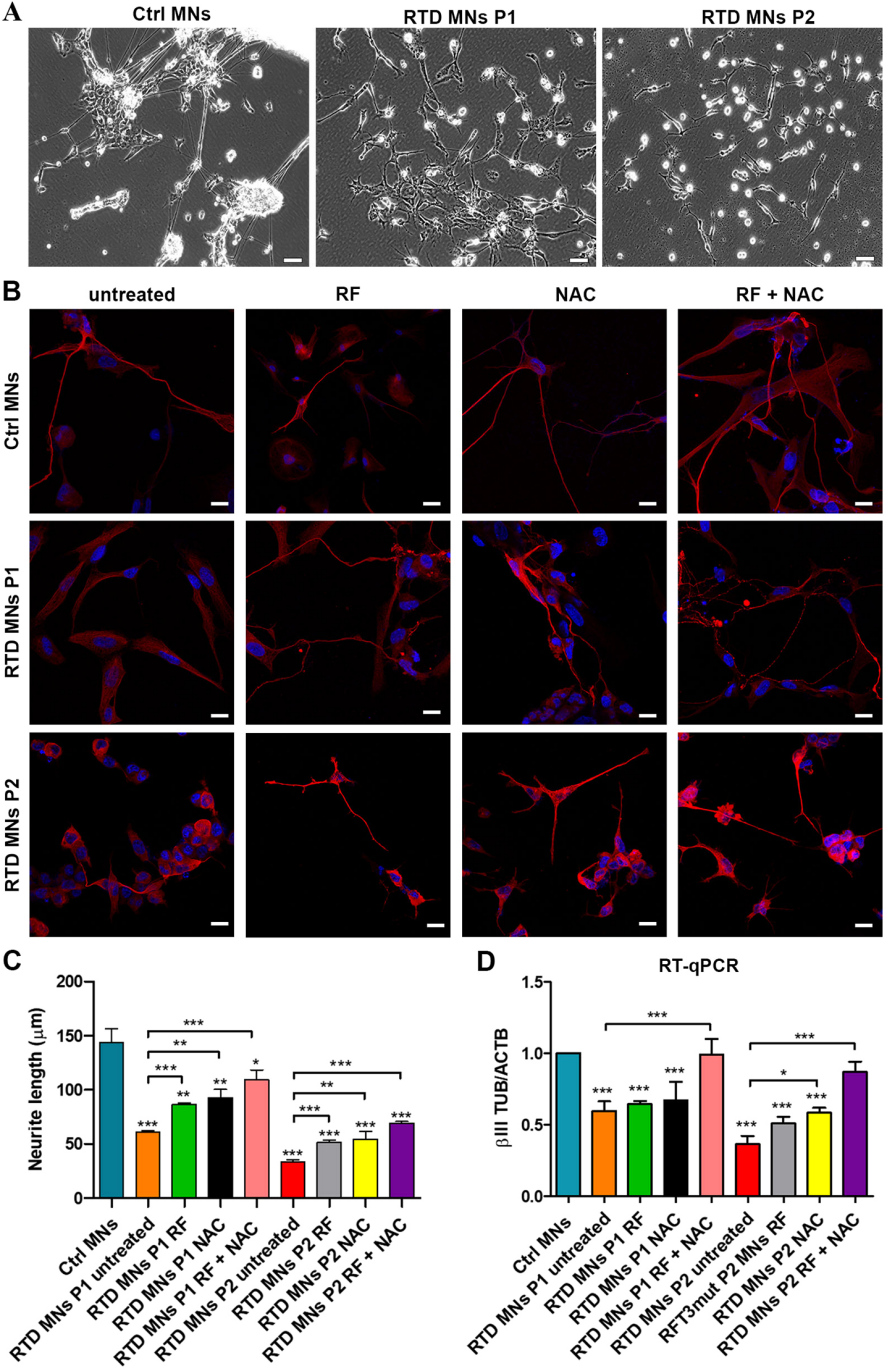
**Altered RTD neuronal morphology is rescued by RF plus NAC treatment.** (A) Phase contrast images showing different morphological features in Ctrl and RTD neurons. Note the presence of shorter neurites can be observed in patient MNs compared to Ctrl MNs, which are characterized by long and well-defined neurites. (B) Immunofluorescence images of βIII-TUB (in red) showing shorter neurites in RTD cells compared to Ctrl. RF or NAC treatment, especially when combined, results in an improvement of general cell morphology and neurite elongation in RTD MNs. Nuclei are stained with Hoechst (in blue). (C) Neurite length analyses of Ctrl and RTD MNs showing significantly shorter neurites in RTD MNs vs Ctrl MNs. Following RF and NAC treatment, especially in combination, a significant increase in neurite length was detected. Data are mean± s.e.m. of three experiments. (D) RT-qPCR of βIII-TUB showing significantly lower levels in RTD than in Ctrl MNs. After RF treatment, βIII-TUB expression increases in patient cells, and the combined RF plus NAC treatment resulted in a further increase of βIII-TUB mRNA levels. *P≤0.05; **P≤0.01; ***P≤0.001; vs Ctrl iPSCs (according to non-parametric ANOVA tests). Scale bars: 20 μm.

To study the cell type mostly affected in RTD patients, we differentiated iPSCs into MNs. Although applying our 30-day-long usual protocol (Corti et al., 2012) to Ctrl iPSCs resulted in regular MNs characterized by the development of well-defined neurites, patient-derived iPSCs differentiated to MNs displaying abnormal features, including the presence of short and immature neurites (Fig. 6A). To get further insights into the morphological and cytoskeletal defects observed in RTD MNs, we examined the localization of βIII-tubulin (βIII-TUB), a specific neuronal marker (Joshi and Cleveland, 1989), which allows the easy identification of neurites and measurement of their length, a fundamental parameter for neuronal maturity during *in vitro* differentiation (Compagnucci et al., 2013). The immunofluorescence signal of βIII-TUB clearly showed shorter neurites in patients' cells compared to Ctrl (Fig. 6B). This morphological defect was partially reversed by either RF or NAC supplementation (Fig. 6B,C). Importantly, the combined RF plus NAC treatment resulted in a substantial improvement of the general cell morphology and neurites' elongation in RTD MNs from both patients (Fig. 6B,C). To quantitatively evaluate the achieved differentiation status of RTD MNs, we measured the length of their neurites. Statistical analysis showed that RTD MNs have significantly shorter neurites compared to Ctrl MNs. Following RF and NAC treatment, especially in combination, a significant increase in neurite length was detected (Fig. 6C).

To get a clearer picture of the overall expression of the *βIII-TUB* gene, we performed RT-qPCR (Fig. 6D), which showed significantly lower levels of βIII-TUB in RTD than in Ctrl MNs. After RF treatment, and even more conspicuously after NAC treatment, βIII-TUB expression increased in the cells of patients. Importantly, the combined RF/NAC treatment resulted in a further increase of βIII-TUB mRNA levels, which reach similar values to those in Ctrl cells (Fig. 6D).

### RTD MNs show impaired calcium intracellular influx

**Fig. 7.**
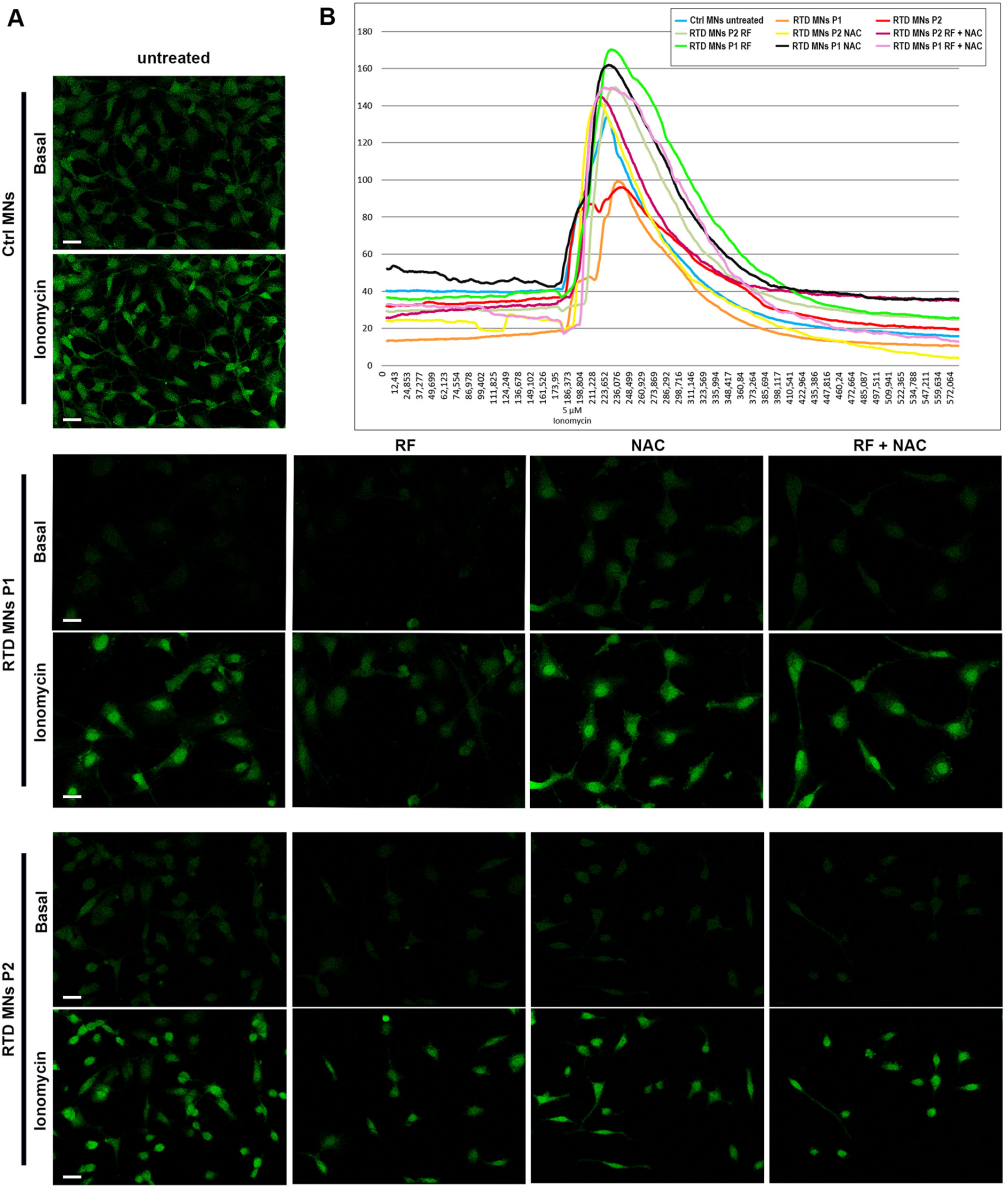
**Impaired calcium intracellular influx in RTD neurons consistently ameliorates following RF and NAC treatments.** (A) Confocal images of calcium assay showing altered Ca^2+^ homeostasis in RTD MNs. The maximal peak of intracellular Ca^2+^ following ionomycin stimulation in RTD MNs was decreased compared to Ctrl MNs. Following RF and/or NAC treatment, peak values of Ca^2+^ levels increased significantly. (B) Graphical representation of the fluorescence mean intensity over time of Ctrl and RTD MNs, showing intracellular Ca^2+^ increase following ionomycin treatment and its decrease following EGTA supplementation in the medium. Scale bars: 20 µm.

To gain further insights into the disease pathophysiology, we performed calcium imaging experiments. Following ionomycin stimulation, MNs functionally respond by triggering calcium influx into the cells and subsequent calcium release from intracellular stores (Fig. 7). Interestingly, the maximal peak of intracellular calcium following ionomycin stimulation in RTD MNs was decreased compared to Ctrl MNs. Importantly, following RF and/or NAC treatment, peak values of calcium levels increased considerably (Fig. 7).

## DISCUSSION

In this study, we took advantage of iPSC technology to address cytoskeletal involvement in the pathogenesis of RTD, a rare genetic disorder characterized by MN degeneration (O'Callaghan et al., 2019). We demonstrated abnormalities in the expression, distribution and arrangement of cytoskeletal components in patient-specific *in vitro* models. These included fibroblast-derived iPSCs and differentiated MNs obtained from two patients, carrying mutations in *SLC52A2*, which encodes the riboflavin transporter RFVT2.

### Morphological and cytoskeletal alterations in RTD iPSCs, and rescue by RF/NAC treatments

The most apparent morphological defect shown by patients' iPSCs relates to their inability to form round-shaped colonies, as opposed to Ctrl cells, and to their widely described *in vitro* behavior (Takahashi and Yamanaka, 2006; Robinton and Daley, 2012; Tokunaga et al., 2014). Because it is known that morphological changes of cultured iPSCs is an important signature to monitor the culture status (Nagasaka et al., 2017), such as the rate and the homogeneity of their undifferentiation status, we were prompted to assess the expression of pluripotency markers *OCT4* and *KLF4* to validate our model (Wakao et al., 2012). Our RT-qPCR results, consistent with our recent report (Colasuonno et al., 2020), confirmed pluripotency of our iPSC model, providing support to the use of iPSCs to study rare genetic disorders. We also assessed cell viability, by MTT test, and demonstrated that no significant changes in the survival rates of iPSCs are associated with RTD. Of note, the MTT assay is based on succinate dehydrogenase activity, thus demonstrating no significant alteration of this mitochondrial flavoenzymatic function in RTD. Thus, despite the abnormal morphology, patients' iPSCs are pluripotent and viable.

As cytoskeletal arrangement is critically implicated in the assembly of cell-cell junctions and the homeostatic regulation of their functions (Vasileva and Citi, 2018), we hypothesized that the above altered cell-cell contacts were related to cytoskeletal defects. In fact, it is conceivable that altered redox status, caused by RF deficiency (Ashoori and Saedisomeolia, 2014; Rizzo et al., 2017; Colasuonno et al., 2020), may affect cytoskeletal structure and arrangement (Wilson and González-Billault, 2015; Islam et al., 2016). Indeed, the significant alterations that we observed in the expression and distribution of MT constituents support this concept. Specifically, decreased signal intensity for α-TUB (P1 and P2) and β-TUB (P2), as well as their abnormal arrangement – concentrated in the perinuclear region, rather than the expected uniform cytoplasmic distribution in a finely reticular form – was detected. Such MT derangement suggests disturbed delivery of adhesion and junctional molecules to the plasma membrane. Among these, E-Cadherin, zonula occludens 1 (ZO-1) and connexin-43 are putative candidates owing to their known interaction with α- and β-TUB (Giepmans et al., 2001). Such impairment is totally consistent with our recent immunofluorescence analysis of RTD iPSCs, showing downregulation and mislocalization of this tight junction marker (Colasuonno et al., 2020), which is known to interact with all the above molecules (Bao et al., 2019; Vasileva and Citi, 2018). Of note, inefficient translocation of E-cadherin to the cell surface has been described in embryonic stem cells lacking proper MT organization (Zenker et al., 2017).

Besides being responsible for the delivery of junctional proteins to cell-cell contact sites, MTs are crucially involved in the intracellular trafficking of organelles, thus indirectly regulating cell homeostasis. It is thus conceivable that disturbances in MT composition and arrangement result in altered energy metabolism. This aspect is especially relevant to our pathological model, in which RF deficiency, per se, impacts the functionality of organelles relying on flavoprotein-directed pathways, namely mitochondria and peroxisomes. The association of peroxisomes and mitochondria to cytoskeleton is widely recognized as an essential and highly regulated process, enabling metabolic efficiency, biogenesis, maintenance and inheritance of these dynamic cellular compartments (Neuhaus et al., 2016; Melkov and Abudu, 2018), particularly those that need long-distance mitochondrial transport. These organelles share an involvement in ROS and lipid metabolism, and susceptibility to oxidative stress, as well as high mobility along the MTs. Indeed, in our previous work we demonstrated peroxisomal and mitochondrial dysfunction in RTD iPSCs, thus relating these alterations to ROS-mediated damage. We reported increased mitochondria-derived superoxide anions, associated with impaired antioxidant response. Such an oxidative stress condition may cause further MT instability, thus exacerbating energy dysmetabolism, in a vicious cycle.

To further investigate MT stability and dynamics, we analyzed the localization of Tyr-TUB and Detyr-TUB in RTD iPSCs, as compared to their Ctrl counterparts. Consistent with the immunofluorescence localization of α- and β-TUB, post-translationally modified MT units are mostly perinuclear in RTD cells, whereas they form a delicate network in Ctrl cells. Importantly, in RTD, P2 Detyr-TUB appeared downregulated, whereas the level of Tyr-TUB was not affected. This suggests an imbalanced Tyr/Detyr ratio, which may account not only for MT instability but even for the impaired neuronal differentiation ability of these cells (see below) (Gadadhar et al., 2017). On the other hand, these results reveal individual-based differences, which in our study also correspond to diverse clinical manifestation. In fact, P2 is characterized by a more severe form of RTD, whereas P1 symptoms are milder. This diversity is found in the morphological defects characterizing iPSCs from the two patients, as well as in β-TUB expression levels, and may reflect a patient-specific ability to cope with metabolic damage caused by RF deficiency.

In this study, we addressed possible beneficial effects of RF treatment on the expression and arrangement of MT components in RTD iPSCs. This proved to be the case, as RF produced an overall increase in α- and β-TUB levels in patients' cells (significant increase in P2). Specifically, Ctrl β-TUB levels were reached by P2 RTD cells, after treatment. Remarkably, the morphological distribution of β-TUB and Detyr-TUB was restored, particularly in P2 iPSCs, which showed fine filaments decorated with both antibodies, following exogeneous RF administration. This prefigures an amelioration in MT arrangement and dynamics, presumably contributing also to the improved mitochondrial functionality reported in our previous work (Colasuonno et al., 2020).

To get a further insight into cytoskeletal organization ability, we evaluated the speed of MT regrowth in timecourse experiments in Ctrl and patients' iPSCs, following administration of nocodazole, a drug that blocks MTs polymerization. Immunofluorescence analyses with α-TUB demonstrate markedly delayed (P2 at 20′ and P1 at 30′) and incomplete re-polymerization, as compared to Ctrl, in which MTs start polymerizing efficiently at 10 min and is apparently completed in 2 h.

As all our data concur in indicating an RTD-associated redox imbalanced state, we performed experiments based on RF treatment, in association with the GSH precursor NAC. Administration of RF or NAC alone results in an earlier start of re-polymerization of α-TUB in RTD cells, but this process remains incomplete. Interestingly, combined RF plus NAC treatment allows full re-polymerization of α-TUB, even in patients' iPSCs within 2 h.

### Altered differentiation and cytoskeletal features in RTD MNs and rescue by RF/NAC treatments

To study the mostly affected cell type in RTD syndrome, Ctrl- and patient-derived iPSCs were differentiated into MNs. Morphological analyses demonstrated structural defects in patients' cells, consisting of shorter (in P1) or hardly recognizable (in P2) neurites, in agreement with the previous study (Rizzo et al., 2017). In contrast, Ctrl MNs showed readily identified and elongated neurites, as expected (Corti et al., 2012).

**Fig. 8.**
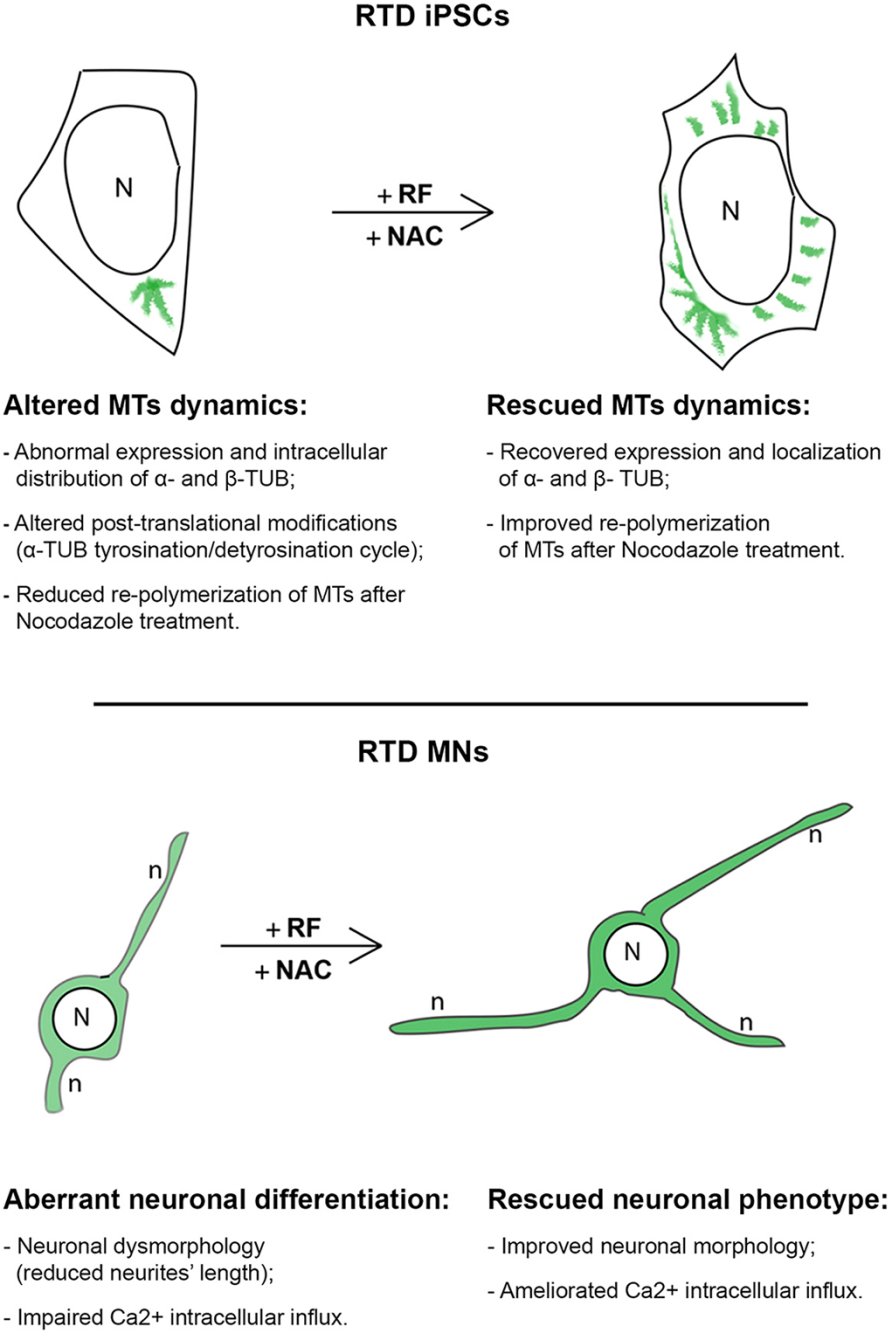
**Synthetic diagram of RTD-associated pathomechanisms involving the cytoskeleton.** The effects of RF and NAC treatments on iPSCs and MNs are summarized. In particular, we depict the altered morphology and MTs in RTD iPSCs, and amelioration of neuronal morphology and calcium influx in RTD neurons following RF plus NAC treatment.

As the cytoskeleton has a primary role in neurite development and extension (Compagnucci et al., 2016), we evaluated the distribution and expression of the neuronal differentiation marker βIII-TUB. Both P1 and P2 MNs displayed reduced βIII-TUB immunopositivity and shorter neurites. We then explored the beneficial effects of RF and NAC, as neuronal integrity is influenced by the redox environment and, consequently, by the GSSG/GSH levels (Piermarini et al., 2016). Even when used alone, RF and NAC produced an improvement in neurite length; even though the most remarkable effect was observed after combined RF plus NAC administration. These data were also confirmed by RT q-PCR analyses, showing that downregulation of βIII-TUB in patients' MNs is rescued by RF plus NAC treatment. Consistent with what was observed for iPSCs, even derived MNs showed individual-based differences, suggesting more severely impaired differentiation of P2 than P1 cells. However, the response to treatments appeared similarly effective in the two cell cultures, supporting administration of RF and NAC for amelioration of the diseased phenotype (Fig. 8).

To gain further insights into the pathophysiology of RFVT2 mutation, we performed calcium imaging experiments in diseased and healthy MNs. Altered response of RTD MNs to ionomycin stimulation was detected, as the maximal peak of Ca^2+^ influx was lower than in Ctrl MNs. Following RF and/or NAC treatment, calcium mobilization increased significantly, reaching Ctrl levels. Calcium imaging measurement is considered a dependable parameter for assessing general neuronal functionality, as Ca^2+^ ions generate versatile intracellular signals that control key functions in all types of neurons (Grienberger and Konnerth, 2012). Furthermore, calcium homeostasis is especially critical in MNs, which rely on it for proper neurotransmission. MNs are more vulnerable than other subtypes to excitotoxicity and dysregulation of intracellular Ca^2+^ homeostasis, as they express high levels of AMPA receptors devoid of the subunit GluR2 enhancing the Ca^2+^ permeability of the AMPA receptor (Williams et al., 1997; Bursch et al., 2019). In another MN disease, namely amyotrophic lateral sclerosis, Ca^2+^ depletion and the accumulation of unfolded proteins was shown to induce endoplasmic reticulum (ER) stress, which affects mitochondria. Even in our RTD syndrome model, it is conceivable that impaired calcium signaling leads to ER stress and contributes to mitochondrial dysfunction, possibly activating autophagic response, in agreement with our previous works (Rizzo et al., 2017; Colasuonno et al., 2020).

### Conclusions

Our study emphasizes the role played by the cytoskeleton in the pathomechanisms of RTD, a childhood-onset MN degenerative disorder. Overall, our data collected on iPSCs and derived MNs argue for multiple damaging effects of RFVT2 mutations, involving structural and functional impairment of the cytoskeleton (Fig. 8). The reason why RF deficiency would affect cytoskeletal arrangement likely relates to oxidative damage to MT components, caused by ROS overproduction, which fails to be counteracted by antioxidant basal defenses (Colasuonno et al., 2020). Although this explanation is supported by solid data (Wilson and González-Billault, 2015; Islam et al., 2016), we cannot rule out the possibility that mutated forms of riboflavin transporters may impair their physical interaction with tubulins, thus contributing to their disorganization. Future research will aim to identify possible direct interactions of RFVTs and specific cytoskeletal components to clarify this issue.

Deranged equilibrium of different forms of MT constituents, although harmful per se, affecting cell morphology and cell-cell contacts, may even impact the functionality of other cytoplasmic compartments, namely mitochondria and peroxisomes (Colasuonno et al., 2020). Thus, energy imbalance, due to insufficient RF supply to flavoprotein-dependent pathways, is exacerbated by altered intracellular trafficking of organelles responsible for ROS and lipid metabolism. This would generate a vicious cycle, which may well account for the rapid progression and the fatal prognosis of the degenerative syndrome. In this context, amelioration of pathological hallmarks, observed after RF and NAC administration, reflects, in our view, the self-sustaining chain of pathomechanisms in such multifaceted disease. Importantly, the proposed combined treatment of the two compounds appears consistently efficacious, irrespective of the severity of disease, suggesting this therapeutic approach to rescue at least in part RTD-associated cell damage. Translational studies hold considerable promise in this respect, as both RF and NAC are easily retrieved, have long-established safety records and do not require titration to achieve the target dose (Back et al., 2020).

In conclusion, our study integrates novel findings with previously reported evidence, providing a framework in which different aspects of the disease connect to each other. If on one hand such crosstalk may account for the severity of the pathology, on the other it provides potential targets to exploit when designing therapeutic strategies.

## MATERIALS AND METHODS

### Derivation of RTD iPSCs

The studies involving human samples were conducted in compliance with the Code of Ethics of the World Medical Association (Declaration of Helsinki) and with national legislation and institutional guidelines (local institutional Ethics Committee of Ospedale Pediatrico Bambino Gesù, Ref 1702_OPBG_2018, date of approval 11 February 2019). Patient and control (Ctrl) fibroblasts were reprogrammed into iPSCs using non-integrating episomal technology (SBI System Biosciences, USA). Ctrl iPSCs were derived from fibroblasts of two healthy individuals. Patients iPSCs were derived from fibroblasts of two RTD patients with *SLS52A2* gene mutation. Informed consent was obtained from all subjects.

### Clinical description of RTD patients

One patient carried the compound heterozygous pathogenic variants c.155C>T (p.S52F) and c.935T>C (p.L312P) (RTD P1), whereas the second affected subject carried the biallelic disease-causing variants c.155C>T and c.1255G>A (p.G419S) (named RTD P2). P1 presented at 3 months of age with macrocytic anemia requiring transfusion and dysphagia. Optic atrophy, axial muscle weakness and respiratory compromise were noted by 1 year, and bilateral sensorineural hearing loss was noted at 2 years. Symptoms, including anemia, were responsive to riboflavin and antioxidant therapy started at 2.5 years of age. P2 was reported in Ciccolella et al. (2013) to develop progressive dysphonia and notable exercise intolerance with dyspnoea and cyanosis at 2 years, bilateral sensorineural hearing loss, reduced visual acuity and progressive shoulder and axial muscle weakness at 3 years. Before his fourth year, P2 required hospitalization for acute respiratory failure and aspiration pneumonia, dying soon thereafter.

### Maintenance of RTD iPSCs and differentiation in MNs

Cells were plated in sixwell plates (Corning, USA) using Matrigel (BD Biosciences, USA), and were maintained using mTeSR Basal Medium (StemCell Technologies, Canada) and 1% penicillin/streptomycin (20 U/ml, 15140122, Thermo Fisher Scientific, Waltham, MA, USA). iPSCs were incubated at 37°C in hypoxic conditions (5% O_2_). The medium was changed every other day.

Differentiation of iPSCs into MNs was performed according to Corti et al. (2012). Cells were plated at a density of 4.2–5.3×10^4^ cells/cm^2^ in NeuroCult NS-A Basal Medium, Human (05750, StemCell Technologies, Canada), for 10 days. Then 0.1 μM retinoic acid (R2625, Sigma-Aldrich) was added to the medium until day 17, when NeuroCult was supplemented with 0.1 μM retinoic acid, 2 μM dorsomorphin (P5499, Merck KGaA, Germany) and 3 ng/ml activin A (SRP3003, Merck KGaA, Germany). On the 24th day, the culture medium was replaced with NeuroCult supplemented with 10 ng/ml brain-derived neurotrophic factor (450-02, Peprotech), 2 ng/ml glial cell-derived neurotrophic factor (450-10, Peprotech) and 200 μM ascorbic acid (A4403, Merck KGaA, Germany). Cells were incubated at 37°C with 5% CO_2_. During the differentiation procedure the medium was changed every other day. General morphological characterization of iPSCs and MNs was assessed using an inverted microscope (Olympus IX 70) equipped with IAS 2000 image capturing software (https://cs.olympus-imaging.jp/en/support/imsg/digicamera/download/software/).

### MTT analysis for cell viability

To measure iPSC viability, an MTT assay (M5655, Merck KGaA, Germany) was conducted. A total of 20,000 cells/well were plated in a 96-well plate and incubated at 37°C for 24 h. The day after, the medium was discarded and 50 µl of MTT (5 mg/ml) was added to each well. Cells were incubated at 37°C for 3 h, then 200 µl of DMSO solvent per well was added. The plate was orbitally shaken in the dark for 15 min, then read with absorbance at OD_570_ nm.

### Drug treatments

Ctrl and RTD iPSCs and iPSC-derived MNs were treated with 10 µM RF (R9504, Merck KGaA, Germany) overnight or 100 µM NAC amide (A0737, Merck KGaA, Germany) overnight. For combined treatment, 10 µM RF and 100 µM NAC were administered together.

### Immunofluorescence analyses

Samples were fixed with 4% paraformaldehyde (PFA) in PBS for 10 min, then treated with the blocking and permeabilizing solution (5% BSA, and 0.1% Triton X-100 in PBS) for 1 h at room temperature. The following primary antibodies and conditions were used: anti-OCT4 monoclonal antibody (MA5-14845, Lot SF2400511A, Thermo Fisher Scientific Inc, USA), 1:400 overnight; SOX2 monoclonal antibody (149811-82, Lot 4310964, Thermo Fisher Scientific Inc, USA), 1:200 overnight; SSEA4 monoclonal antibody (MA1-021, Lot SB247948, Thermo Fisher Scientific Inc, USA), 1:250 overnight; TRA-1-60 (SC21705, Lot B2117, Santa Cruz Biotechnology Inc, USA), 1:100 overnight; anti-α-tubulin monoclonal antibody (T5168, Lot 038M4813V, Merck KGaA, Germany), 1:500 for 2 h at room temperature; anti-β-tubulin polyclonal antibody (2146S, Lot No 9, Cell Signaling Technology, USA), 1:100 overnight at 4°C; anti-α-tubulin tyrosined monoclonal antibody (Tyr-TUB, MAB1864-I, Lot 3319821, Merck KGaA, Germany), 1:500 for 2 h at room temperature; anti-α-tubulin detyrosinated antibody (Detyr-TUB, AB48389, Lot GR19854, Abcam), 1:200 overnight at 4°C; anti-hRFVT1 polyclonal antibody (GTX87668, Lot 821401795, GeneTex Inc, USA), 1:50 overnight at 4°C; anti-hRFVT3 polyclonal antibody (GTX51591, Lot 821700752, GeneTex Inc, USA), 1:50 overnight at 4°C; anti-hRFVT2 polyclonal antibody (GTX87976, Lot 821604261, GeneTex Inc, USA), 1:50 overnight at 4°C; and the neuronal marker anti-β III-tubulin monoclonal antibody (T2200, Lot 059M4891V, Merck KGaA, Germany), 1:500 for 2 h at room temperature. The immunoreaction was revealed by appropriate secondary antibodies conjugated with Alexa Fluor 488 or Alexa Fluor 555 (Thermo Fisher Scientific), diluted 1:500 in PBS for 1 h at room temperature. Nuclei were stained with 1 µg/ml Hoechst (33342, Thermo Fisher Scientific) at 1:10,000 in PBS for 10 min at room temperature. The coverslips were mounted using 1:1 PBS/glycerol.

### SDS-PAGE and western blot analyses

For western analysis, cells were lysed in RIPA buffer (50 mM Tris-HCl, 1 mM EDTA, 1 mM EGTA, 150 mM NaCl and 1% NP-40) supplemented with complete protease inhibitor cocktail (Roche). Proteins were separated by SDS-PAGE and transferred to nitrocellulose membrane. Membranes were blocked in 5% milk for 1 h at room temperature. Primary antibodies were blotted overnight at 4°C. Secondary antibody-horseradish peroxidase conjugates were blotted for 1 h at room temperature and membranes stained with SuperSignal West Pico Chemiluminescent Substrate (Pierce Biotechnologies). The following primary antibodies were used: α-TUB (Sigma-Aldrich, Cod. T5168, Lot 038M4813 V), 1:10,000 overnight; β-TUB (Sigma-Aldrich, Cod T4026, Lot No 9), 1:10,000 overnight; and GAPDH (Abcam, Cod ab8245, Lot 6C5), 1:10,000 overnight.

### Microtubule re-polymerization analyses

Ctrl and RTD iPSCs were treated with 10 μM nocodazole (M1404, Merck KGaA, Germany) and incubated at 37°C for 35 min. After nocodazole removal, MT regrowth was analyzed in timecourse experiments. When considering RF- or/and NAC-treated cells, supplementation was performed 24 h before the nocodazole treatment. Cells were first fixed with 4% PFA, then processed for α-TUB immunofluorescence and Hoechst staining, as described above.

### Neurite length measurement

To measure neurite length of Ctrl and RTD MNs, images were acquired using a Leica DMi8 inverted microscope equipped with a Leica DFC 450C camera. Quantitative evaluations were made using the LAS X software (www.leica-microsystems.com/products/microscope-software/p/leica-las-x-ls/) on β-III tubulin-stained neurites as described by Compagnucci et al. (2013). Only neurons with soma and processes that were completely included in the captured images were analysed. At least 100 neurites of Ctrl and RTD MNs were analyzed from three independent experiments.

### qRT-PCR

Total RNA from iPSCs and iPSC-derived MNs of healthy and diseased individuals was isolated using Trizol Reagent (Merck KGaA, Germany), according to the manufacturer's protocol, and quantified using a Nanodrop 2000 Spectrophotometer (Thermo Fisher Scientific). For each sample analyzed, 1 μg of total RNA was reverse transcribed using Euro Script Reverse Transcriptase (Euroclone, Italy) and RT Random Hexamers as primers (Euroclone, Italy). RT-qPCR was used to evaluate the expression levels of pluripotency markers (*OCT4* and *KLF4*) in iPSCs and neuronal markers (*β-III-TUB*) in MNs. The PCR reaction was carried out with Power SYBR Green dye chemistry (Thermo-Fisher Scientific) using the ABI PRISM 7500 Fast Real-Time PCR System (Thermo Fisher Scientific). Data were analyzed using the 2-Delta-Delta Ct method with *ACTIN B* (*ACTB*) as the housekeeping gene.

### Calcium imaging

For calcium imaging, the procedure by Glaser and colleagues (2016) was followed. iPSC-derived MNs were grown in 35 mm optical plates (Ibidi, Germany) previously coated with Matrigel, and washed with Hank's balanced salt solution (Thermo Fisher Scientific). The live probe Fluo-4 (Cod F10489, Thermo Fisher Scientific) was used according to manufacturer's instruction. After registering the baseline signal for 3 min, 5 μM ionomycin (Thermo Fisher Scientific) was added to the cells. The maximum peak of fluorescence was recorded, and after 30 s, 30 mM EGTA (SLBR7504V, Merck KGaA, Germany) was added. The recordings were carried out for a total of 10 min. The live acquisition was conducted with a frame rate of 2 frames per second, 20× magnification with a 1024×800 format and an electronic zoom at 2.0, using a TCS-SP8X Leica confocal microscope equipped with the 8000 Hz resonant module and a stage incubator (OkoLab, Naples, Italy), allowing temperature to be maintained at 37°C and humidity maintained at 5% CO_2_. For each biological replicate, 10-20 cells were measured. Lines in the diagrams represent the normalized average fluorescence intensity change over time. For quantification, the area under the curve of the whole Fluo-4 fluorescence peak area was determined using GraphPad Prism.

### Confocal microscopy

Confocal optical sectioning was performed with a Leica TCS-SP8X microscope equipped with a white light laser source and a 405 nm diode laser. Sequential confocal images were acquired using a HC PLAPO 63× oil immersion objective (1.40 numerical aperture, Leica Microsystems, Germany). Moreover, samples were thoroughly photographed at 20× magnification to allow the quantitative evaluation of immunofluorescence intensity with the LAS X analysis module. Representative images were assembled using Adobe Photoshop CS6 software (Adobe Systems Inc, USA).

### Statistical analysis

Statistical analysis was performed on data obtained from at least three independent experiments in triplicate using the GraphPad Prism 7.0 Software. Statistically significant differences were analyzed using one-way ANOVA between columns, following tests assessing the normal distribution of data. The data are presented as as mean±s.e.m. Statistical significance was defined as *P<0.05, **P<0.005 and ***P<0.001. The bar graphs reporting no asterisk are not significant according to non-parametric ANOVA tests.

## Supplementary Material

Supplementary information
