# Supplementary material for: Altered cytoskeletal arrangement in induced pluripotent stem cells and motor neurons from patients with riboflavin transporter deficiency
Source: Dis Model Mech. 2021 Feb 24;14(2):dmm046391. doi: 10.1242/dmm.046391 (PMC7927654; doi:10.1242/dmm.046391)
Supplement: Supplementary information [file dmm-14-046391-s1.pdf]

Suppl Material

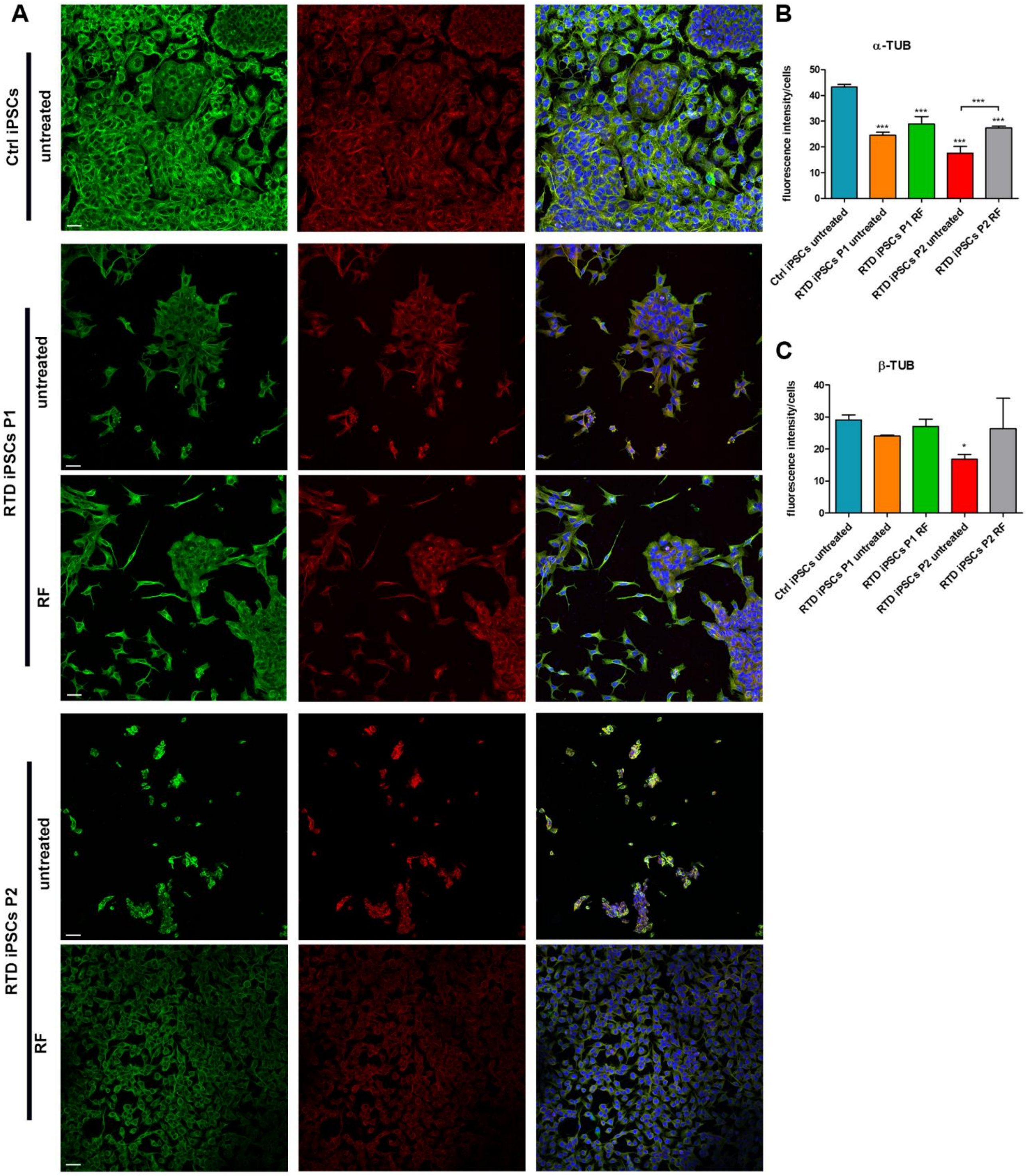

Fig. S1. Immunofluorescence localization of α-TUB (in green) and β-TUB (in red), demonstrating different intracellular distribution in RTD iPSCs, as compared to Ctrl cells. (A) Representative images at original magnification 20x, on which our statistical analysis was performed. Scale bars: 20 µm. (B) Bar graph of α-TUB fluorescence intensity and statistical analysis revealing significantly lower levels of α-TUB in both RTD patients' iPSCs (****P*≤0.001) versus Ctrl iPSCs. Note increased intensity levels after RF treatment. (C) Bar graph of β-TUB fluorescence intensity and statistical analysis revealing abnormally low levels in P2 cells (**P*≤0.05). Data are mean±s.e.m.
